# Complete genome sequence of *Burkholderia contaminans* PCR-C_Y9 isolated from *Pericarpium citri reticulatae* 'Chachiensis'

**DOI:** 10.1128/mra.01230-24

**Published:** 2025-02-12

**Authors:** Fan Xu, Yongkai Ma, Gu Chen

**Affiliations:** 1 School of Food Science and Engineering, South China University of Technology 26467 , Guangzhou, China; Portland State University, Portland, Oregon, USA

**Keywords:** *Burkholderia contaminans* PCR-C_Y9, *Pericarpium citri reticulatae* 'Chachiensis', lipid degradation

## Abstract

*Burkholderia contaminans* PCR-C_Y9 was isolated from *Pericarpium citri reticulatae* ‘Chachiensis’ (PCR-C), the natural aged pericarps of *Citrus reticulata* Blanco ‘Chachiensis.’ It could degrade PCR-C lipids. Its genome was sequenced as 8.70 Mbp with a guanine–cytosine content 66.23%. It was annotated to understand properties and possible contribution to aging of PCR-C.

## ANNOUNCEMENT

*Pericarpium citri reticulatae* (PCR) is the dried and aged pericarps of *Citrus reticulata* Blanco and its cultivars, while PCR-Chachiensis (PCR-C) is the most premium one (1). They have been used as medicine and food additive for hundreds of years (1), and we have revealed the central bacterial community and identified probiotic bacterial strains from PCR-Chachiensis (2, 3). Screening the lipid-degrading microbe in PCR-C is an interesting topic for further characterization. *Burkholderia contaminans* PCR-C_Y9 was isolated from PCR-C collected from an orchard in Xinhui District in Guangdong, China through screening on basic salt culture medium [(NH₄)₂SO₄ 2.38 g/L, KH₂PO₄ 1.36 g/L, CaCl₂·2H₂O 6.63 mg/L, MgSO₄·7H₂O 0.25 g/L, Na₂HPO₄ 1.42 g/L, FeSO₄·7H₂O 2.8 mg/L, 2.5 ‰ Triton X-100 (v/v), 1.5% agar (m/v)] supplemented with 1% lipid (m/v), which was extracted from PCR-C through Soxhlet methods with petroleum ether (4). Clear and transparent hydrolysis cycle around PCR-C_Y9 colony indicated degradation of PCR-C lipid. PCR-C_Y9 was identified as *Burkholderia contaminans* through 16S rRNA gene sequencing (5).

PCR-C_Y9 DNA was extracted from an overnight lysogeny broth shaking culture through DNA extraction following an initial washing step with STE buffer (0.25 M sucrose, 0.03 M Tris, 0.05 M EDTA) (6). Whole-genome sequencing was performed by Nanopore PromethION and Illumina NovaSeq. To construct 10 kb library for Nanopore, large fragments of DNA were recovered by using a BluePippin automatic nucleic acid fragment recovery system, and barcode was added by using an EXP-NBD104 Kit (Oxford Nanopore, UK). Afterwards, the SQK-LSK109 Kit was used to link the adapter. Library for Illumina NovaSeq was generated using NEBNext Ultra DNA Library Preparation Kit (NEB, USA) after sonification of DNA to a size of 350 bp. Raw nanopore data attained from Nanopore flow cell R10.4.1 was filtered with NanoPlot v1.29.1 (threshold Q > 7), and Illumina data were filtered with Trimmomatic v0.40 (Table 1). Default parameters were used for all software unless otherwise specified.

**TABLE 1:** Information on sequencing data statistics

Illumina NovaSeq		Nanopore PromethION	
Statistical item	Value	Statistical item	Value
Raw paired reads	4,839,150	Raw reads	279,342
Raw base (Mbp)	1,452	Raw base (Mbp)	1,556
Insert size (bp)	350	Clean reads	267,737
Clean reads length (bp)	150:150	Clean base (Mbp)	1,483
Clean data (Mb)	1,341	Mean read length (bp)	5,541
Scaffolds total depth	131	N_50_ read length (bp)	8,868

Unicycler v0.4.8 (7) was used to combine Illumina and Nanopore data for genome assembly. The assembled 8.70 Mbp PCR-C_Y9 genome comprised three circular chromosomes and two circular plasmids. The sizes for chromosomes 1, 2, and 3 and plasmids 1 and 2 were 1.53, 3.25, and 3.61 Mbp and 0.24 and 0.07 Mbp, respectively. Their guanine–cytosine content was 65.78, 66.71, and 66.42% and 61.99 and 58.96%, respectively. NCBI Prokaryotic Genome Annotation Pipeline v6.8 (8) was applied to annotate the genome as 7,723 protein-coding sequences, 18 rRNA, and 69 tRNA genes. SignalP v6.0 (9) and DeepTMHMM v1.0.42 was used to predict secretory protein. Among the 545 secretory proteins predicted, four phospholipase, two glycerophosphodiester phosphodiesterase, and two lipase were annotated. Neighboring to the secreted triacylglycerol lipase gene is the gene predicted to encode lipase secretion chaperone. Meanwhile, six secreted alpha/beta fold hydrolases might contribute to the lipid-degrading capacity of PCR-C_Y9 since they contain amino acid sequences conserved in esterase as the catalytic site in a three-dimensional structure predicted by AlphaFold 3 (10, 11). Availability of the *Burkholderia contaminans* PCR-C_Y9 genome sequence will allow further investigation into the lipid degrading properties of this bacterium and its contribution to the aging process of high-quality PCR-C.

## Data Availability

The whole-genome sequence of *Burkholderia contaminans* PCR-C_Y9 has been deposited at GenBank under accession numbers CP172005, CP172006, CP172007, CP172008, and CP172009 with the BioSample accession number SAMN44367217 and the BioProject accession number PRJNA1175622. The raw sequence reads have been submitted to the Sequence Read Archive under accession numbers SRR31277720 and SRR31277721.
